# Do Socio-Economic Characteristics Affect Travel Behavior? A Comparative Study of Low-Carbon and Non-Low-Carbon Shopping Travel in Shenyang City, China

**DOI:** 10.3390/ijerph15071346

**Published:** 2018-06-27

**Authors:** Jing Li, Kevin Lo, Meng Guo

**Affiliations:** 1 Northeast Institute of Geography and Agroecology, Chinese Academy of Sciences, Changchun 130102, Jilin, China; 2 Department of Geography, Hong Kong Baptist University, Kowloon Tong, Hong Kong, China; lokevin@hkbu.edu.hk; 3 School of Geographical Science, Northeast Normal University, Changchun 130024, Jilin, China; guom521@nenu.edu.cn

**Keywords:** travel behavior, socio-economic characteristics, shopping mobility, influencing factors, China

## Abstract

Choices regarding mode of travel have an evident effect on environment pollutants and public health. This paper makes a significant contribution by examining the differences between low-carbon and non-low-carbon travel mode choices during shopping trips, and how socio-economic characteristics impact individual travel behavior based on data gathered from a questionnaire conducted in Shenyang, China. The study found that, firstly, low-carbon travel modes were more common than non-low-carbon travel modes for shopping, and the average travel distance by non-low-carbon modes was a little longer than that of low-carbon modes. Secondly, suburban and wholesale specialized commercial centers attracted more residents travelling longer distances by non-low carbon modes, especially private car, compared to regional commercial centers in inner city areas. Thirdly, strong relationships between car ownership, gender, monthly income, and travel mode choices were identified in a binary logistic regression model. This study thus highlights the importance of sustainable transportation policies to advocate low-carbon travel modes and reduce carbon emissions.

## 1. Introduction

There is clear scientific agreement that carbon emissions are affecting the global climate with irreversible long-term consequences [1]. Mitigating energy-related emissions plays a key role in the future of sustainable urban development [2]. The transport sector, as the largest and fastest growing energy consuming sector and contributor to environmental externalities, is inseparably linked to the climate-change challenge; it is currently responsible for 23% of total energy-related GHG emissions [3,4,5]. Furthermore, it is predicted that China’s transport carbon emissions will continue to grow rapidly and will contribute about one-third of global CO_2_ emissions by 2030 [6,7]. In developed Western cities, it is widely believed that the automobile has provided the means for cities to spread, leading to extensive suburbanization, longer travel distances and low population densities. However, according to the latest data from the World Health Organization (WHO), nearly 7 million people die from air pollution each year, a large extent of which is caused by automobile exhaust emissions. With the affluent middle classes buying cars as soon as they can afford to, motor vehicle ownership and utilization are growing rapidly in China [8]. According to the China Statistical Yearbook 2017, the number of small private passenger cars nationwide rose from 10.8 million to 146.5 million from 2005 to 2016, with an average annual growth rate of 26.7%. With the recent surge in the numbers of private vehicles, reducing energy consumption in the transport sector and lowering the adverse effects of environmental pollutants on public health is regarded as an urgent priority in urban China presently [9].

The travel behavior of urban residents is of increasing interest to researchers and planners. Choices of travel mode for daily mobility have significant effects on the future development of urban regions due to urban sprawl and structural changes to growing metropolitan areas related to transportation development [10,11,12,13,14]. A particularly noteworthy trend in China is the increase in shopping trips, which exacerbate traffic congestion, environmental pollution, and public health issues [15,16]. The function of commercial centers is to supply goods and services to meet the needs and desires of the public [17]. In many advanced countries, one of the most marked changes in the spatial retail structure of cities since the beginning of the 20th century is decentralization [16,18]. On the one hand, China’s metropolises are following the same path, with suburban shopping malls, large supermarkets, and new formats to serve surrounding suburban residents [19,20]. On the other, the commercial and service sectors remain in city centers and maintain a strong accumulation effect, especially in municipal commercial districts. In the context of this situation, the layout of commercial centers further directly influences transport mode choices and environmental externalities during shopping trips [21,22,23,24,25].

Previous studies that have analyzed low-carbon development in the transport sector mainly focused on technological improvement strategies and policies to reduce carbon emissions [7,8,26,27]. Zheng et al. [28] proposed an integrated policy for decreasing the use of urban light-duty vehicles, improving fuel economy and promoting electric vehicles and biofuels to assure peak national vehicle GHG emissions are reached in China by approximately 2030. De Gennaro et al. [29], in a study endorsed by the European Council, stated that key areas for reducing greenhouse gas emissions in the transport sector are switching towards carbon-free or less carbon-intensive fuels and improving fuel efficiency. However, shifting toward less destructive modes of public transport and active travel has been proposed as another important strategy for achieving a significant reduction in carbon emissions from transportation, as technological innovation alone does not suffice [30,31,32]. Consequently, a large body of research on travel behavior has emerged which is directed toward developing more informed insights for policymakers [33,34,35,36]. The identification of factors influencing transport mode choice is particularly important for proposing effective climate change mitigation policies and strategies [37,38]. Extensive literature has discussed the links between the built environment and travel behavior [39,40,41,42,43]. In general, empirical studies have identified associations between the characteristics of the built environment such as density, street design, land use diversity, destination accessibility, distance to transit, and demographics, and travel behavior [37,44,45,46,47]. However, empirical studies on built environment influences cannot fully resolve the details of individual travel behavior, which can also be impacted by the socio-economic characteristics of individuals [48,49]. An important aspect of our study is to provide understanding through a modal shift towards low-carbon travel behavior alternatives capable of mitigating transport carbon emissions and negative environmental impacts of public health. Hence, a key question we focus on is: to what extent do personal socio-economic characteristics influence travel behavior by replacing the traditional built environment and travel behavior relationship approach? Previous studies on travel behavior mainly focused on daily commuting patterns [50,51,52,53]. However, there is still little evidence of non-working travel, especially for shopping trips, and travel behavior has become more complex due to the layout of commercial centers and rapid expansion of urban sprawl. Within this context, this paper aims to examine the differences between low-carbon and non-low-carbon travel mode choices for shopping trips, and how socio-economic characteristics impact individual travel behaviors within the city of Shenyang, one of the largest metropolitan areas in China.

## 2. Materials and Methods

### 2.1. Study Area

**Figure 1:**
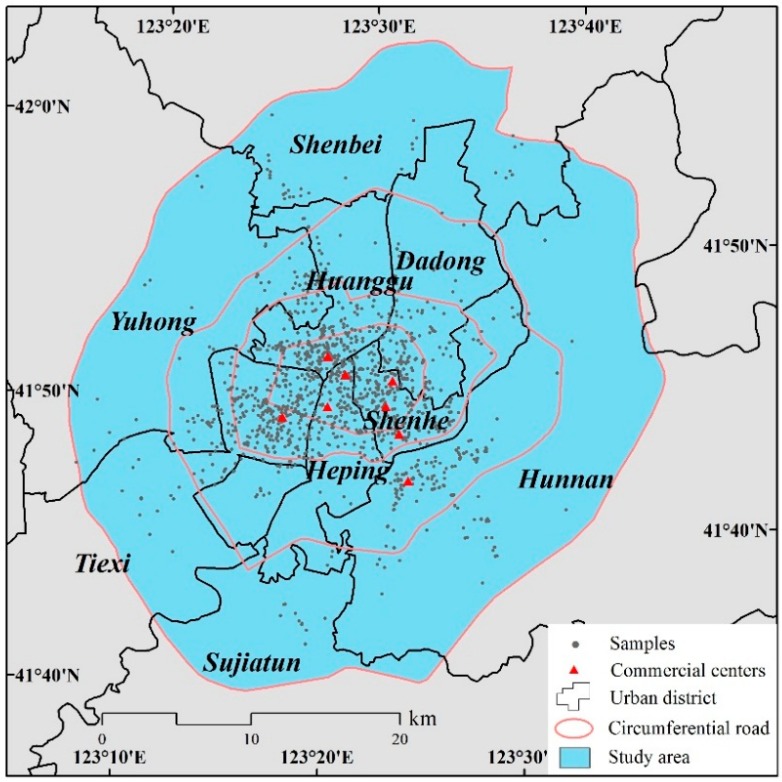
Map of study area.

Shenyang city, located in the middle of Liaoning Province, is the provincial capital and the largest city in northeast China [54]. It contains nine urban districts: Heping, Shenhe, Dadong, Huanggu, Tiexi, Sujiatun, Hunnan, Yuhong, and Shenbei. There is a total area of 1232 square kilometers and 3.77 million people in the central urban area of Shenyang. Shenyang has been a city of heavy industry since the early 1900s, and is undergoing rapid economic redevelopment through new construction in the Shenyang metropolitan area [55,56]. Today, tertiary industries account for 42.5% of GDP. Commercial services have been greatly improved, with retail sales of consumer goods reaching 363.61 billion RMB in 2015. Eight typical commercial centers were selected as our field sites based on their location characteristics and types of retail present [57]. Middle Street and Taiyuan Street are two municipal commercial centers at the city center, with a large business circle covering the entire municipality of Shenyang, and even extending to surrounding cities. The commercial centers of Beihang, Xita-Beishi, and Tiexi are regional shopping centers located in the inner city, which mainly serve residents of the surrounding areas. Wuai and Nanta are specialized wholesale markets with retail activities. Hunnan commercial center, located in the suburbs, has gradually developed with the construction of the Hunnan New Area (Figure 1).

### 2.2. Data Collection

The data used in this study were collected through questionnaire surveys and interviews conducted with residents on shopping trips to eight typical commercial centers on weekends and weekdays. The survey consisted of three parts: one about travel behavior such as travel mode, travel time, frequency of trips and travel routes; another on personal socio-economic characteristics such as car ownership, gender, age, education, occupation, and monthly income; and a third on attitudes towards the development of public transport and their opinions on shopping by private car. A random sample of 1672 shoppers was invited to respond to the questionnaire, which was filled out by 1525 people, a 91.21% response rate.

### 2.3. Methodology

Logistic regressions have been widely used to identify the determinants of travel behavior in many contexts [58,59,60,61,62]. The transport mode data in our study were categorized as the binary response variables, ‘low-carbon transport mode’ (walking/cycling, electric bike, bus and metro) and ‘non-low-carbon transport mode’ (private car and taxi), and a binary logistic regression model was adopted to examine the major determinants of socio-economic characteristics of travel behavior during shopping trips. The binary logic model for this study allows for the prediction of binary outcomes (a value of 1 with a probability of *p* for the respondents’ travel decisions of non-low-carbon transport mode and a value of 0 with probability 1-*p* for choosing low-carbon transport mode), using one or more continuous or categorical variables of socio-economic characteristics as predictors [63,64]. The binary logistic regression model can be written as Equation (1) [65,66].

(1) $$\ln\left[\frac{\hat{Y}}{1-\hat{Y}}\right]=\beta_{0}+\sum_{r=1}^{n}\beta_{r}x_{r}$$

where $\hat{Y}$ is the probability of the outcome variable being equal to 1 (choosing non-low-carbon transport mode for shopping), *β*_0_ is the model constant, and *x_r_* is a continuous or categorical predictor variable. Parameter *β*_r_ is the regression coefficient to be estimated.

## 3. Results and Discussions

### 3.1. Respondent Socio-Economic Characteristics

It is necessary to sum up the socio-economic characteristics of respondents surveyed in the eight typical commercial centers (Table 1). The results indicated that 36% of the 1525 respondents owned private cars. Regarding gender, females constituted 62.6% of the sample. Younger respondents were more likely to go shopping, especially those 35 years of age or younger, who made up 63.0%. The majority of respondents had received tertiary education (59.1%). A high proportion of the workforce in the survey sample (35.9%) was in business fields. Respondents earning income between 3000 and 5000 CNY per month accounted for the largest percentage of 30.8%.

### 3.2. Travel Behavior of Respondents during Shopping Trips

The mode choice behavior of respondents for shopping trips to the eight typical commercial centers was shown in Table 2. Overall, low-carbon travel modes (80.5%) were more frequent than non-low carbon modes for shopping. With regard to the specific mode of transport, nearly half the respondents preferred to go shopping by bus, 17.3% preferred to walk or cycle and 14.1% preferred to travel by private car. Average travel distance by non-low-carbon modes was slightly higher than that of low-carbon modes, which affected the total carbon emissions produced. Of all commercial centers, respondents travelling by non-low-carbon modes, especially private car, to Hunnan commercial center accounted for the largest proportion (27.5%). The average travel distance was 7.53 km. As Hunnan commercial center is located in the low-density suburbs with better road conditions and sufficient parking facilities, it attracts many residents driving private cars for shopping. From this, it could be suggested that large-scale suburban shopping centers increase city sprawl and induce more traffic [67]. The wholesale specialized commercial centers of Wuai and Nanta saw respondents travelling the longest distances, approximately 9 km by private car or taxi; this may be due to its relatively low wholesale prices. The municipal commercial centers of Middle Street and Taiyuan Street in the city center witnessed relatively long travel distances of 7.58 km and 7.03 km using non-low-carbon modes. An interesting finding was that there was no decline in the central business district, which had a strong contingent of both local and regional shoppers. Nevertheless, Carling et al. [68] indicated that the majority of shopping activity has moved from downtown to edge-of-town, such that shopping activity there is now 10 times higher than that downtown. Hence, in many developed countries, suburban shopping centers have come to dominate the retail structure in metropolitan areas, resulting in the deterioration of Central Business Districts [18]. As the inner city regional commercial centers of Xita-Beishi, Beihang, and Tiexi mainly serve residents in the closely-surrounding areas, most residents prefer to travel to these regional commercial centers by low-carbon transport modes, with the exception of Xita-Beishi, a specialty business street including flower markets, birds, fish, art, and antiques. Travel distances by low-carbon modes were comparatively short, approximately 4.20 km, 5.16 km, and 5.19 km on average to Xita-Beishi, Beihang and Tiexi, respectively. Interestingly, it is worth noting that there is a larger proportion of shoppers walking and cycling to these centers: 38.68%, 30.77%, and 23.65% in Xita-Beishi, Beihang, and Tiexi, respectively.

### 3.3. Impacts of Socio-Economic Factors on Transport Mode Choice

To test content validity, we developed a binary logistic regression model to explain how socio-economic factors affect transport mode choices during shopping trips. Socio-economic factors included gender, age, education level, occupation, and monthly income. The estimation results of the binary logistic regression model analysis for the choice between non-low-carbon and low-carbon transport mode are presented in Table 3. We found that the conclusions drawn regarding the effects of transport modes were based on models of modest statistical fit (Chi^2^ = 270.220; *df* = 15; *p* = 0.000), although their explanatory power was low (Nagelkerke R^2^ = 0.256). Using a significance test, we found the socio-economic variables of car ownership and gender to be significant at a 99.9% confidence level, and monthly income significant at a 99% confidence level. This may explain the increase in the use of non-low-carbon transport modes for shopping.

As with findings from previous studies, this study confirms car availability has the largest impact on the choice of non-low carbon transport modes for residents during shopping trips. The vehicle ownership results indicate that residents having their own vehicles were 5.629 times more likely to select non-low carbon modes for shopping trips than those who do not own a car. Plaut [69], Choi and Ahn [70], and Carse et al. [71] have also shown that car ownership is strongly associated with a reduced likelihood of choosing non-motorized modes of transportation, and the higher the number of cars available per adult in the household, the more likely that the trip would be made by non-low-carbon transport modes. Hence, reducing dependency on cars, including through the control of vehicle ownership and vehicle use intensity, improving public transport and non-motorized infrastructure and services, and developing new, cleaner fuel and fuel-efficient vehicles could be more effective strategies to reduce energy consumption, GHG emissions, and the adverse effects of environmental pollution in Chinese cities [7,72,73]. It was observed that males were 1.928 times more likely to use non-low-carbon modes for shopping than females. This finding further emphasized the role of gender differences, which was reflected in previous research. Cao [74] and Myers et al. [75] demonstrated a strong association between gender and travel mode choice. Women were significantly less likely than men to choose non-low-carbon modes, and female respondents interviewed in our study stated that they were more likely to use public transport, especially on days with bad weather or poor road conditions. Income is also an important driver of mode choice in travel behavior. From our data and analysis, we found that residents with monthly incomes of less than 2000 CNY, 2000–3000 CNY and 3000–5000 CNY were 0.421 times, 0.522 times and 0.588 times more likely to choose non-low-carbon modes during shopping trips, respectively, than residents with monthly income higher than 5000 CNY. Along with rising personal disposable incomes and an expanding middle class buying private cars as soon as they can afford to, private automobile ownership and usage are increasing, while public transport usage has almost universally declined in China in the past decades [76,77].

## 4. Conclusions

This study was performed by obtaining data from questionnaire surveys on weekends and weekdays in Shenyang, China, to explore low-carbon and non-low-carbon travel mode choice behavior during shopping trips to eight typical commercial centers, examining how socio-economic characteristics impact travel patterns and carbon emissions. Our study shows that low-carbon travel modes are more frequent than non-low-carbon modes for shopping. Furthermore, travel mode choice is associated with the location and retail type of the commercial centers. Suburban and specialized wholesale market-oriented commercial centers attract many residents travelling longer distances by non-low-carbon modes of transportation, especially private cars, for shopping. The municipal commercial centers of Middle Street and Taiyuan Street in the city center witness relatively long travel distances by non-low-carbon modes. The regional commercial centers of Xita-Beishi, Beihang, and Tiexi, in the inner city, attract shoppers who almost always choose low-carbon modes, and for whom the travel distances are shorter. The results of binary logistic regression indicate that car ownership, gender, and monthly income are significantly associated with travel mode choices.

The findings of the study have some implications for sustainable transport and low-carbon urban planning. First, they suggest that there should be a shift of emphasis from expanding transport networks to policies that encourage the use of low-carbon alternatives. According to previous research, the improvement of low-carbon transport infrastructure and the adoption of different strategies should depend on city size [3]. Notably, enhancing a better quality public transport system, especially bus services, should be prioritized in the metropolitan areas of China’s megacities to lower the use of non-low-carbon transport. It will be important for appropriate transport policies to be formulated and enhanced concerning accessibility, seat availability, reliability, affordability, comfort, and safety of public transport infrastructure and services. An equally important policy measure is addressing the spatial configuration of different types of retail centers, which is crucial in achieving the aims and goals of low-carbon urban planning, as supported by earlier findings [68]. Especially in the development of regional commercial centers surrounded by high-density housing, efficient public transport systems and good infrastructure for cycling and walking have effectively led to a reduction in travel distances, improved low-carbon travel modes, and reduced environmental pollution. More importantly, urban design policy should seek to encourage people with different socio-economic characteristics who have a strong preference for non-low-carbon transport modes to adopt sustainable travel behavior, and to raise environmental awareness. Finally, the implication is that, combined with technological investment into the development of new cleaner-fuel and fuel-efficient vehicles, sustainable low carbon transportation transport policies should be put in place as part of behavioral interventions, including car and fuel taxes, road pricing, and congestion charging. Parking constraints and parking fees are of particular priority to Hammadou and Papaix [78], who found that parking management policies are significant in shifting to low-carbon transport modes and reducing the adverse effects of air pollution on public health.

## Figures and Tables

**Table 1:** Socio-economic characteristics of respondents (%).

Car Ownership	Gender	Age Group	Education	Occupation	Monthly Income
Yes (36.2) No (63.8)	Male (37.4) Female (62.6)	≤18(1.97) 19–25(28.13) 26–35(34.82) 36–50(18.49) ≥51(16.59)	Below High school (26.16) High school (14.75) Undergraduate (54.75) Above Master (4.33)	Public (15.34) Business (35.93) Self-employed (17.51) Unemployed and retirement (31.21)	<2000 CNY (15.00) 2000–3000 (28.30) 3000–5000 (30.80) >5000 (25.90)

**Table 2:** Travel behavior data for shoppers at eight commercial centers.

Commercial Center	Low-Carbon Mode								Non-Low-Carbon Mode			
	Walking/Cycling		Electric Bike		Bus		Metro		Private Car		Taxi	
	Proportion (%)	Distance (km)	Proportion (%)	Distance (km)	Proportion (%)	Distance (km)	Proportion (%)	Distance (km)	Proportion (%)	Distance (km)	Proportion (%)	Distance (km)
Wuai	6.53	2.12	3.52	6.81	66.33	8.17	2.51	12.40	13.07	12.42	8.04	6.94
Nanta	17.35	2.35	1.53	5.43	62.76	8.29	1.02	8.25	12.76	11.24	4.59	6.78
Hunnan	11.76	2.65	0.49	6.30	30.88	9.43	29.41	13.98	20.10	8.27	7.35	6.79
Middle Street	15.38	2.08	0.45	5.60	45.25	8.54	19.91	11.20	15.38	8.52	3.62	6.64
Taiyuan Street	6.77	2.31	0.52	4.30	41.15	8.82	28.13	8.13	15.63	7.64	7.81	6.42
Xita-Beishi	38.68	1.35	2.83	3.83	32.08	7.11	0.94	4.50	22.64	5.86	2.83	4.70
Beihang	26.94	1.76	1.37	2.33	55.25	6.06	1.83	10.95	10.50	6.22	4.11	5.47
Tiexi	23.65	1.66	3.45	5.06	47.48	7.00	13.79	7.03	6.90	5.82	4.43	3.38

**Table 3:** Logistic regression results of impacts on transport mode choice during shopping trips.

Explanatory Factors	B	S.E.	Wals	Exp (B)	95% C.I. for Exp (B)	
					Lower	Upper
Car ownership (ref: no)	1.728 ***	0.158	119.745	5.629	4.131	7.671
Gender (ref: female)	0.657 ***	0.145	20.488	1.928	1.451	2.563
Monthly income(ref: >5000 CNY)						
Monthly income (<2000)	−0.866 **	0.303	8.154	0.421	0.232	0.762
Monthly income (2000–3000)	−0.650 **	0.207	9.872	0.522	0.348	0.783
Monthly income (3000–5000)Age (ref: ≥51)	−0.530 **	0.170	9.777	0.588	0.422	0.820
Age (≤18)	−0.312	0.536	0.340	0.732	0.256	2.093
Age (19–25)	0.003	0.280	0.000	1.003	0.579	1.737
Age (26–35)	0.375	0.267	1.979	1.455	0.863	2.455
Age (36–50)Occupation (ref: retirement and unemployed)	0.342	0.280	1.492	1.407	0.813	2.435
Occupation (public)	0.204	0.245	0.693	1.226	0.759	1.981
Occupation (business)	0.092	0.210	0.192	1.096	0.726	1.655
Occupation (self-employed)Education (ref: above master)	0.106	0.230	0.214	1.112	0.709	1.745
Education (below high school)	0.142	0.426	0.111	1.153	0.500	2.655
Education (high school)	0.629	0.382	2.707	1.876	0.887	3.970
Education (undergraduate)	0.359	0.351	1.050	1.432	0.720	2.848
Constant	−2.815 ***	0.455	38.227	0.060		
Pseudo R-Square (Nagelkerke)	0.256					
−2 Log Likelihood	1251.411					
Chi-Square	270.220					

*** *p* < 0.001; ** *p* < 0.01; * *p* < 0.05.
